# A Comparison of Accelerometer Cut-Points among Individuals with Coronary Artery Disease

**DOI:** 10.1371/journal.pone.0137759

**Published:** 2015-09-11

**Authors:** Stephanie A. Prince, Jennifer L. Reed, Amy E. Mark, Christopher M. Blanchard, Sherry L. Grace, Robert D. Reid

**Affiliations:** 1 Division of Prevention and Rehabilitation, University of Ottawa Heart Institute, Ottawa, Ontario, Canada; 2 Institute for Clinical Evaluation Services, Ottawa, Ontario, Canada; 3 Department of Medicine, Dalhousie University, Halifax, Nova Scotia, Canada; 4 School of Kinesiology and Health Science, York University, Toronto, Ontario, Canada; 5 Toronto Rehabilitation Institute, University Health Network, Toronto, Ontario, Canada; Irvine, UNITED STATES

## Abstract

**Background:**

Accurate assessment of physical activity among coronary artery disease patients is important for assessing adherence to interventions. The study compared moderate-to-vigorous physical intensity activity and relationships with cardiometabolic health/fitness indicators using accelerometer cut-points developed for coronary artery disease patients versus those developed in younger and middle-aged adults.

**Methods:**

A total of 231 adults with coronary artery disease wore an Actigraph GT3X accelerometer for ≥4 days (≥10 hours/day). Moderate-to-vigorous intensity physical activity between cut-points was compared using Bland-Altman analyses. Partial spearman correlations assessed relationships between moderate-to-vigorous intensity physical activity from each cut-point with markers of cardiometabolic health and fitness while controlling for age and sex.

**Results:**

Average time spent in bouts of moderate-to-vigorous intensity physical activity using coronary artery disease cut-points was significantly higher than the young (mean difference: 13.0±12.8 minutes/day) or middle-aged (17.0±15.2 minutes/day) cut-points. Young and middle-aged cut-points were more strongly correlated with body mass index, waist circumference and systolic blood pressure, while coronary artery disease cut-points had stronger relationships with triglycerides, high-density and low-density lipoproteins. All were similarly correlated with measures of fitness.

**Conclusion:**

Researchers need to exert caution when deciding on which cut-points to apply to their population. Further work is needed to validate which cut-points provide a true reflection of moderate-to-vigorous intensity physical activity and to examine relationships among patients with varying fitness.

## Background

Cardiovascular diseases, including coronary artery disease (CAD), are the leading cause of mortality globally [1–3]. Importantly, CAD outcomes are largely dependent on gains in cardiometabolic fitness achieved via exercise interventions (e.g. cardiac rehabilitation) [2–7]. Regular physical activity (PA) is an important contributor to cardiometabolic fitness in patients with CAD [5, 8, 9]. The American College of Sports Medicine recommends patients with CAD accumulate 30 to 60 minutes of moderate-to-vigorous intensity (40–80% $\dot{\text{V}}$O_2peak_) PA (MVPA) on most, but preferably all, days of the week [10].

Accurate assessment of PA among CAD patients is important to: effectively monitor whether patients are meeting PA guideline recommendations; assess relationships between PA and markers of health and health events; and, for evaluating the effectiveness of interventions. Research has shown that objective and self-report measures of PA do not capture the same information, with self-reports often over-estimating levels compared to objective measures [11]. Objective measures such as accelerometers overcome these limitations by offering a more accurate measurement of PA (frequency, duration, intensity) and energy expenditure [12]. Accelerometers provide a continuous measure of the frequency and amplitude of movement to generate a “count” with larger counts indicative of higher intensities of PA.

To date, accelerometer (device-specific) count cut-points to differentiate PA intensities have largely been established using apparently healthy adult populations [13, 14]. However, relative activity count cut-points have been shown to differ based on cardiorespiratory fitness levels [15]. For instance, CAD patients entering cardiac rehabilitation often present with compromised cardiorespiratory function likely as a result of the disabling effects of the coronary event and the de-conditioning during recovery [16]. Therefore, the use of accelerometer cut-points developed in apparently healthy, high-functioning individuals may result in a misclassification of PA intensity amongst these individuals.

Recently, Mark et al. (unpublished, presented below) proposed the first known PA intensity cut-points for use in high functional capacity CAD populations. While these population-specific cut-points have been suggested, they have yet to be evaluated for their appropriateness in quantifying PA among individuals with CAD, and have not been validated against bio-markers of cardiometabolic health and fitness (i.e. lipids, body mass index [BMI], $\dot{\text{V}}$O_2peak_). Further, their degree of agreement with widely applied cut-points established in apparently healthy adult populations remains uncertain. Therefore, the objectives of this study were to examine the agreement in MVPA levels using accelerometer cut-points developed for CAD patients with those developed in healthy younger [14] and middle-aged adults [17], and to compare associations with established indicators of cardiometabolic health and fitness.

## Methods

This study was conducted at two sites: the University of Ottawa Heart Institute (UOHI) and University Health Network (UHN) in Ontario, Canada. Three sets of previously-proposed accelerometer cut-points for identifying MVPA (Mark (unpublished), Sasaki [14], Santos-Lozano [17]) were compared using raw accelerometer counts from cardiac patients enrolled in an ongoing randomized controlled trial (RCT). This research received ethical approval from the Ottawa Health Science Network Research Ethics Board (UOHI #201200579–01, #2011139-01H, #20130276-01H) and the UHN Research Ethics Board (UHN 12-5018-AE9l). All participants provided written informed consent prior to participating.

### Participants

Adults with complete baseline data (to date) were selected from a RCT (ECologically Optimizing exercise maintenance in men and women Post-Cardiac Rehabilitation [ECO-PCR]) evaluating the efficacy of an exercise facilitator-lead intervention on long-term exercise maintenance among patients who have completed cardiac rehabilitation in comparison to usual care. Additional study details have been described elsewhere (http://clinicaltrials.gov/ct2/show/NCT01658683).

### Accelerometers

Baseline accelerometer results available from all participants recruited to date for the ECO-PCR trial were used for this study. Participants were asked to wear an ActiGraph GT3X accelerometer (ActiGraph, Pensacola, FL) on their right hip during waking hours for nine days. Participants were instructed to remove the monitor while sleeping or during water-related activities (e.g. swimming or bathing). The GT3X accelerometer is a lightweight and compact triaxial accelerometer that can capture movement about the vertical (y-axis), horizontal (x-axis) and perpendicular (z-axis) axes. It also provides output for the vector magnitude; a composite measure of all three axes (vector magnitude = √(x^2^ + y^2^ + z^2^)). A 15-second sampling epoch was used and converted into counts-per-minute (cpm).

### Accelerometer data reduction

A valid day was defined as ≥10 hours of wear time, and participants were required to have a minimum of four valid days to be retained in the analyses [18]. For participants with more than seven valid days, the first day was removed (minimize reactivity) and the subsequent seven days used for the average. Wear time was calculated by subtracting non-wear time from 24 hours. Non-wear time was defined as at least 60 minutes of consecutive zeros for counts, with an allowance of up to two minutes of counts between zero and 150. Minutes spent in bouts (≥10 consecutive minutes) were used to quantify MVPA for seven days in line with the World Health Organization’s PA recommendations [19]. Weekly average was calculated by multiplying the daily average (minutes/day) by seven.

### Accelerometer cut-points

Three sets of cut-points developed for the ActiGraph GT3X using vector magnitude were applied to this cohort of participants [14, 17]. These cut-points were compared with regard to their classification of individuals as meeting PA guidelines or not (≥150 minutes/week of MVPA), and for their associations with markers of cardiometabolic health and fitness. Table 1 provides a comparison of the three sets of cut-points employed for this analysis.

**Table 1:** Cut-point characteristics.

	Vector magnitude cut-points		
	CAD patients (Mark) N = 18	Healthy young (Sasaki [14]) N = 36	Healthy middle-aged(Santos-Lozano [17]) N = 31
**Study population**			
Females, n (%)	1 (6%)	unknown	15 (48%)
Age (years)	58.3 ± 10.7	28.0 ± 9.0	47.1 ± 3.5
$\dot{\text{V}}$O_2_ (mL/kg/min)	29.5 ± 7.6†	≥31.5‡	≥23.4‡
Body mass index (kg/m^2^)	28.4 ± 5.6	23.7 ± 3.3	23.5 ± 2.9
**Physical activity intensity cut-point ranges**			
Moderate (cpm)	1800–3799	2690–6166	3208–8564
Vigorous (cpm)	≥3800	≥6167	≥8565

Data presented as mean ± standard deviation unless otherwise specified.

†$\dot{\text{V}}$O_2peak_

‡ maximum average $\dot{\text{V}}$O_2peak_ reached on a sub-maximal test

CAD–coronary artery disease, cpm–counts-per-minute

#### Mark cut-points

The Mark cut-points were based on a convenience sample of predominantly male, older (45–80 years), and high functional capacity (mean $\dot{\text{V}}$O_2peak_ = 29.3 ± 7.4 mL/kg/min) CAD patients enrolled in cardiac rehabilitation. Participants were recruited if they had an exercise stress test ordered as part of routine care. A progressive treadmill exercise test with a ramp protocol was employed to establish cpm cut-points for moderate and vigorous intensity PA based on the corresponding relative exercise intensity (moderate: 45–59% $\dot{\text{V}}$O_2peak_, vigorous: ≥60% $\dot{\text{V}}$O_2peak_) measured by oxygen uptake using a metabolic cart.

The authors employed two methods to determine the cut-points. First, a mixed-model approach was used to model the relationship between cpm and relative PA intensity (e.g. 45% of maximum aerobic capacity = moderate intensity) to determine cut-points for moderate and vigorous intensity PA. Secondly, a Receiver Operator Characteristic (ROC) curve analysis was used to plot the sensitivity and specificity of the cut-points from the mixed models, as well as higher and lower counts min^-1^ values. The model and ROC curve analysis resulted in cut-points of 1800–3799 cpm for moderate and ≥3800 cpm for vigorous intensity PA.

#### Sasaki cut-points [14]

The Sasaki cut-points were based on a young volunteer population of 36 participants (males and females). The established cut-points were: 2690–6166 cpm for ‘moderate’ intensity; 6167–9642 cpm for ‘hard’ intensity; and, >9642 cpm for ‘very hard’ intensity.

#### Santos-Lozano cut-points [17]

The Santos-Lozano cut-points were based on a middle-aged (40–55 years) volunteer population of 16 and 15 apparently healthy men and women, respectively. The cut-points included: 3208–8564 cpm for ‘moderate’; 8565–11592 cpm for ‘vigorous’; and, ≥11593 cpm for ‘very vigorous’ intensity.

### Markers of cardiometabolic health

In addition to the accelerometer results, baseline data were extracted for several cardiometabolic markers of health to assess their relationships with time spent in bouts of MVPA from the cut-points. Markers included: measured height and weight to determine BMI in kg/m^2^, measured waist circumference, systolic and diastolic blood pressure (BP), HbA1c percentage, triglycerides, total cholesterol, high-density lipoprotein (HDL), and low-density lipoprotein (LDL).

### Markers of cardiorespiratory fitness

A random sub-sample of participants (n = 140) completed a symptom-limited graded exercise test with electrocardiographic monitoring using a ramp protocol on a treadmill to obtain $\dot{\text{V}}$O_2peak_ (mL kg min^-1^) as an indicator of cardiorespiratory fitness. Exercise tolerance was also assessed among all participants using the Duke Activity Status Index (DASI), a self-administered questionnaire that measures functional capacity and an estimate of an individual's peak oxygen uptake [20]. Finally, self-reported weekly MVPA was assessed using a modified version of the Godin Leisure-Time Exercise Questionnaire [21].

### Statistical analysis

All analyses were conducted using SAS version 9.3 (SAS Institute Inc., Cary, NC, USA). All summary data were tested for normality using plots and Shapiro-Wilk’s test statistics. Descriptive data were reported using means ± standard deviation (SD) or frequencies and percentages. Mean values between males and females for descriptive data were compared using the Wilcoxon-Mann-Whitney test.

Mean values between the three cut-points for total time spent in moderate and vigorous intensity PA, as well as time spent in bouts of 10 minutes or more of MVPA were compared using paired Wilcoxon signed rank sum tests, linear regression and Bland-Altman analyses. The proportion of those meeting versus not meeting MVPA guidelines between the three cut-points was compared using a chi-square analysis. Partial spearman correlations were used to assess the relationships between time spent in bouts of MVPA (wear time-adjusted) from each cut-point with each of the cardiometabolic health and fitness indicators while controlling for age and sex. Adjustments were made for age and sex as cardiorespiratory fitness levels are known to decline with age and are known to be more strongly linked with risk factors and cardiovascular disease among males than females [22, 23]. MVPA was adjusted for wear time by using the residuals produced by regressing MVPA bout time on total wear time [24].

## Results

Out of 241 eligible participants, 231 (182 males, 49 females) with valid accelerometer results were used for the analyses. The majority (79%) had seven valid days of data. Results were similar regardless of whether missing days were imputed based on daily averages or when only using those with seven days of valid data.

Table 2 provides participant characteristics. On average, participants were considered overweight, were normotensive, appeared to have well-managed lipids, and had a moderate-to-high functional capacity [25, 26].

**Table 2:** Participant characteristics.

Characteristic	Total (N = 231)
**Sociodemographic**	
Age (years)	63.7 ± 9.2 (36–85)
Males, n (%)	182 (79%)
**Medications**	
Beta-blockers, n (%)	180 (78%)
Statins, n (%)	207 (90%)
**Clinical**	
Body mass index (kg/m^2^)	28.1 ± 4.6 (17.8–44.8)
Waist circumference (cm) [n = 230]	99.2 ± 12.6 (64.6–143.0)
Systolic blood pressure (mm Hg) [n = 229]	120 ± 18 (57–175)
Diastolic blood pressure (mm Hg) [n = 230]	73 ± 10 (47–113)
HbA1c (%) [n = 213]	5.9 ± 0.7 (4.6–8.9)
Total cholesterol (mmol/L) [n = 227]	3.43 ± 0.86 (1.50–8.56)
HDL (mmol/L) [n = 227]	1.16 ± 0.31 (0.60–2.40)
LDL (mmol/L) [n = 226]	1.70 ± 0.63 (0.20–5.10)
Triglycerides (mmol/L) [n = 227]	1.26 ± 0.69 (0.40–7.90)
DASI score [n = 184]	47.3 ± 11.4 (19.0–65.7)
$\dot{\text{V}}$O_2peak_ (mL/kg/min) [n = 140]	25.4 ± 7.0 (7.6–52.0)
Self-reported MVPA (min/day) [n = 194]	29.4 ± 25.7 (0–162.9)
Accelerometer steps (number/day)	7211 ± 2768 (1469–15836)

Data presented as mean ± standard deviation and (min–max) unless otherwise specified.

DASI–Duke Activity Status Index, HDL–high density lipoprotein, LDL–low density lipoprotein, MVPA–moderate-to-vigorous intensity physical activity

Average time spent in bouts of MVPA using the Mark cut-points was significantly higher than MVPA quantified using the Sasaki cut-points (mean difference: 13.0 ± 12.8 minutes/day, p < .0001) or the Santos-Lozano cut-points (mean difference: 17.0 ±15.2 minutes/day, p < .0001) (see Table 3). MVPA from the cut-points was highly correlated (Mark/Sasaki *r* = 0.91, p < .0001 and Mark/Santos-Lozano *r* = 0.84, p < .0001).

**Table 3:** Physical activity levels by cut-point.

	Cut-points		
Physical activity intensity	CAD population (Mark)	Healthy young (Sasaki [14])	Healthy middle-age(Santos-Lozano [17])
Moderate, minutes/day	62.7 ± 31.1	39.4 ± 21.9	31.9 ± 21.7
Vigorous, minutes/day	23.6 ± 19.5	4.7 ± 9.9	0.4 ± 1.7
MVPA, minutes/day	86.3 ± 40.5	44.1 ± 26.1	32.3 ± 22.2
MVPA, minutes/week†	603.8 ± 283.1	308.9 ± 182.3	225.8 ± 155.6
**MVPA bouts, minutes/day**	**37.9** ± **26.4**	**24.9** ± **19.8** *	**20.9** ± **18.4** *
**MVPA bouts, minutes/week** †	**265.1** ± **184.1**	**174.3** ± **138.6** *	**146.2** ± **128.6** *
Percentage meeting 150 minutes/week	71%	50%	43%

Data presented as mean ± standard deviation unless otherwise specified.

CAD–coronary artery disease, MVPA–moderate-to-vigorous intensity physical activity

*significantly different from the Mark cut-points

†Based on daily average multiplied by 7

**Fig 1:**
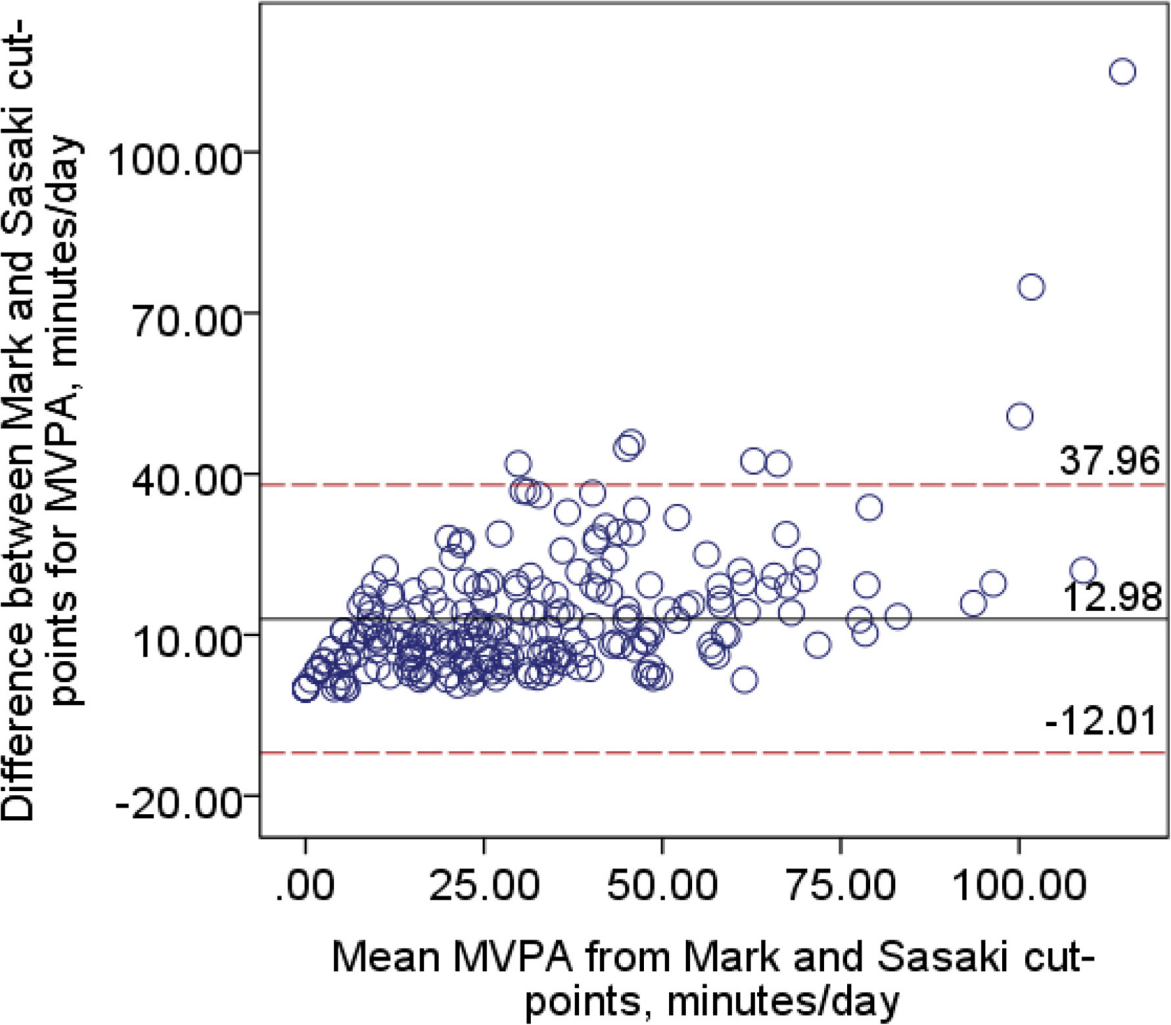
Bland-Altman plot for minutes per day spent in bouts of MVPA between the Mark and Sasaki [14] cut-points.

**Fig 2:**
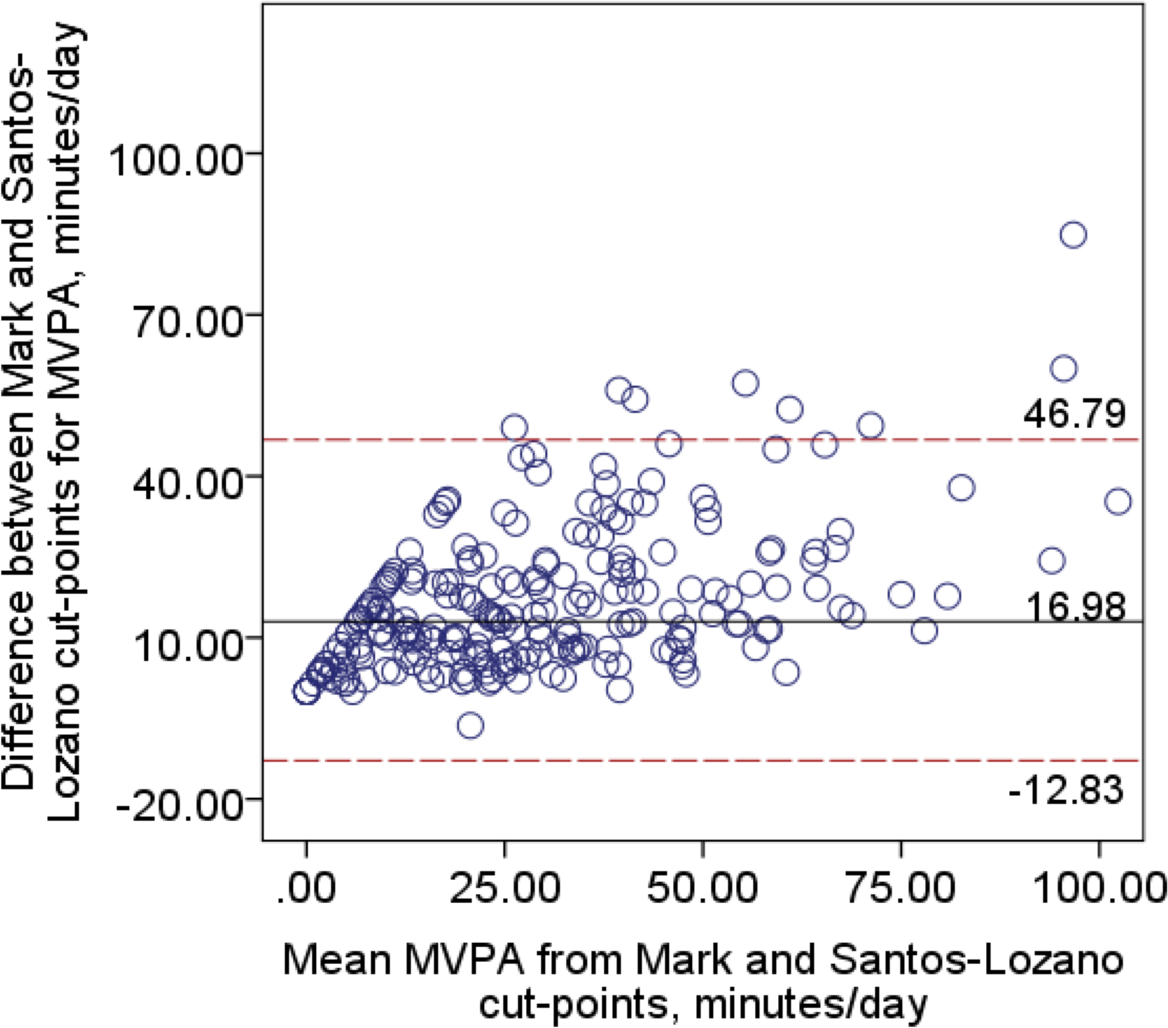
Bland-Altman plot for minutes per day spent in bouts of MVPA between the Mark and Santos-Lozano [17] cut-points.

Limits of agreement showed poor agreement between the cut-points and ranged from -12.0 to 38.0 minutes/day (Fig 1) when comparing the Mark and Sasaki cut-points, and from -12.8 to 46.8 minutes/day (Fig 2) when comparing the Mark and Santos-Lozano cut-points. Results of the linear regression analyses revealed a significant proportional bias (t_move-sasaki_ = 9.51, t_move-santoslozano_ = 9.97, p < .0001), suggesting that with greater amounts of MVPA, the disagreement between the cut-points increased. The Mark cut-points resulted in a significantly greater proportion of participants classified as having met the MVPA guidelines of ≥150 minutes/week of MVPA compared to the Sasaki or Santos-Lozano cut-points (p < .0001).

As shown in Table 4, the Sasaki and Santos-Lozano cut-points were more strongly correlated with BMI, waist circumference and systolic BP than the Mark cut-points. The Mark cut-points had stronger relationships with triglycerides, HDL and LDL, though with LDL not in the expected direction. With regard to cardiorespiratory health, all three cut-points had similar correlations with $\dot{\text{V}}$O_2peak_, self-reported MVPA and DASI score (trend only with Sasaki).

**Table 4:** Partial spearman correlations between MVPA (wear-time adjusted) and markers of cardio-metabolic health by cut-point, controlling for age and sex.

	Cut-points		
Marker of cardio-metabolic health	CAD population (Mark)	Healthy young (Sasaki [14])	Healthy middle-age (Santos-Lozano [17])
Body mass index	*-0*.*12*, *p = 0*.*07*	**-0.17, p = 0.01**	**-0.18, p = 0.006**
Waist circumference	**-0.13, p<0.05**	**-0.17, p = 0.01**	**-0.16, p = 0.01**
HbA1c	-0.06, p = 0.43	-0.06, p = 0.42	-0.06, p = 0.38
Systolic blood pressure	*-0*.*11*, *p = 0*.*10*	**-0.17, p = 0.01**	**-0.14, p = 0.04**
Diastolic blood pressure	0.03, p = 0.63	-0.003, p = 0.96	0.03, p = 0.61
Total cholesterol	*0*.*11*, *p = 0*.*09*	0.03, p = 0.64	0.03, p = 0.65
Triglycerides	-0.11, p = 0.11	-0.09, p = 0.21	-0.09, p = 0.21
HDL	**0.15, p = 0.02**	*0*.*11*, *p = 0*.*09*	*0*.*12*, *p = 0*.*06*
LDL	**0.13, p<0.05**	0.05, p = 0.47	0.03, p = 0.63
$\dot{\text{V}}$O_2peak_ (n = 140)	**0.28, p = 0.0008**	**0.31, p = 0.0003**	**0.32, p = 0.0001**
Self-reported MVPA (n = 185)	**0.25, p = 0.0007**	**0.29, p<0.0001**	**0.33, p<0.0001**
DASI score	0.12, p = 0.11	0.11, p = 0.14	*0*.*13*, *p = 0*.*07*

DASI–Duke Activity Status Index, HDL–high density lipoprotein, LDL–low density lipoprotein, MVPA–moderate-to-vigorous intensity physical activity

**Bolded** results are statistically significant, *italicized* results approach statistical significance

## Discussion

The results of the study indicate a substantial difference in the derived daily minutes engaged in bouts of MVPA among the Mark, Sasaki and Santos-Lozano cut-points. Although MVPA from all three cut-points was strongly related, there was a significant lack of agreement between the cut-points. The difference was most apparent when using the data for categorizing those meeting current MVPA guidelines; with the CAD specific cut-points categorizing a greater majority of participants as achieving the guidelines. MVPA derived from all three cut-points were similarly related, with moderate correlations observed between MVPA (in bouts) and $\dot{\text{V}}$O_2peak_ and self-reported MVPA.

Regular MVPA is recognized as beneficial for producing health benefits including reducing the risk for cardiometabolic diseases and premature mortality [7, 27]. Moderate-to-vigorous intensity PA is also important for the secondary prevention of recurrent cardiovascular disease amongst those with established CAD. Since individuals with CAD often present with compromised cardiorespiratory functioning which can affect relative activity count cut-points [15], we had hypothesized that the Mark cut-points would show stronger associations with $\dot{\text{V}}$O_2peak_ and markers of cardiometabolic disease than other frequently used cut-points developed in apparently healthy and likely higher functional capacity individuals [14, 17]. When the cut-points were compared, the differences in continuous time spent in bouts of MVPA between the three cut-points were fairly substantial (13–17 minutes/day) and resulted in the CAD-specific cut-points classifying a greater proportion of participants as having achieved the recommended 150 minutes/week of MVPA. The differences between cut-points tended to be higher when individuals were more active. These findings have significant implications for both researchers and clinicians who use the guidelines as a means of identifying individuals at risk for future heart disease and events. The application of Mark’s cut-points could ultimately result in a reduced emphasis on continuing to increase daily PA. However, given that the current study’s population, although diseased, had a high functional capacity and had just completed cardiac rehabilitation, the results of the Mark cut-points are plausible. This is especially true given that the Mark cut-points were developed using a population that was enrolled in cardiac rehabilitation with higher fitness levels than participants in the current study (mean $\dot{\text{V}}$O_2peak_ 29.5 vs. 25.4 mL/kg/min). It is therefore feasible that the higher proportion meeting MVPA guidelines using the Mark cut-points is more reflective of the ‘true’ proportion meeting the guidelines.

The relationships between continuous minutes of MVPA and cholesterol measures were stronger with the Mark cut-points. The partial correlations identified that as MVPA increased, triglycerides decreased and HDL and LDL increased. Literature has consistently documented that MVPA is related to HDL and triglyceride values, but not LDL [28–30]. Further, the LDL findings are also likely explained by the large number of participants taking statins for lowering cholesterol, specifically LDL [31].

All three cut-points showed either no significant or low correlations with the cardiometabolic markers aside from $\dot{\text{V}}$O_2peak_. Moderate-to-vigorous intensity PA was moderately correlated with $\dot{\text{V}}$O_2peak_ using all three cut-points. This is not surprising given that PA is one of the strongest predictors of cardiorespiratory fitness [12]. The low or lack of correlations with the other variables may be partially explained by the minimal variability among the various markers within this study population. On average, participants were considered overweight and exceeded waist circumference guidelines [32], but were normotensive, appeared to have their lipids well-managed (within Canadian guideline target lipid values [33, 34]), and had a relatively high functional capacity [26]. It is also possible that participants were engaging in a greater amount of MVPA due to a reactive response from wearing the monitors, and the MVPA results reported herein are not typical. The majority of participants were on optimal medical therapy. It is likely that because the risk factors in this group were adequately controlled, we would need a much larger sample size to increase variability in order to see stronger linear relationships between increasing MVPA quantities and these indicators. Further a prospective cohort design is needed to verify whether reductions in these risk factors varied by cut-point applied.

This study is not without limitations. Firstly, all three cut-points were developed using treadmill protocols that involved walking or running in a controlled laboratory setting. Participants in the current study wore the accelerometers in free-living situations where not all of the minutes of MVPA would have been accumulated by walking or running, such as in the case of cycling, aerobics classes or swimming. It is thus possible that these cut-points may lead to some misclassification of MVPA achieved. On the other hand, it is important to note that walking is the most commonly reported PA behavior [35] and is the most used mode of exercise for cardiac rehabilitation [36]. Second, the population in this study on average is classified as having a high functional capacity and was comprised of recent cardiac rehabilitation graduates; therefore, the findings herein are not necessarily generalizable to CAD patients who have not undergone such PA intervention or whose risk factors are not well controlled. Finally, this was a cross-sectional study and therefore no causal conclusions can be drawn.

## Conclusions

This study is the first to compare accelerometer cut-points developed for use in a clinical CAD population with others developed in healthy young and middle-aged adult populations. Results showed that the cut-points are not comparable when looking at time spent in continuous bouts of MVPA. The results showcase the need for clinicians, researchers, health and fitness professionals and industry to exert caution when deciding on which cut-points to apply to their population. Ultimately further validation work is needed to identify which cut-points provide a better reflection of those achieving PA recommendations in CAD patients. Further research would benefit from reproducing the CAD cut-point validation study in a larger cohort of average CAD patients (not immediate post-cardiac rehabilitation), as well as testing the cut-points among female and low functional capacity CAD patients.
